# Delivering Optimal Care to People with Cognitive Impairment in Parkinson's Disease: A Qualitative Study of Patient, Caregiver, and Professional Perspectives

**DOI:** 10.1155/2023/9732217

**Published:** 2023-08-29

**Authors:** Jennifer S. Pigott, Nathan Davies, Elizabeth Chesterman, Joy Read, Danielle Nimmons, Kate Walters, Megan Armstrong, Anette Schrag

**Affiliations:** ^1^Queen Square Institute of Neurology, University College London, London, UK; ^2^Centre for Ageing Population Studies, Research Department of Primary Care and Population Health, University College London, London, UK

## Abstract

**Background:**

Cognitive impairment is common in Parkinson's disease (PD) and associated with lower quality of life. Cognitive impairment in PD manifests differently to other dementia pathologies. Provision of optimal care requires knowledge about the support needs of this population.

**Methods:**

Eleven people with PD and cognitive impairment (PwP), 10 family caregivers, and 27 healthcare professionals were purposively sampled from across the United Kingdom. Semistructured interviews were conducted in 2019–2021, audio-recorded, transcribed, and analysed using reflexive thematic analysis.

**Results:**

Cognitive impairment in PD conveyed increased complexity for clinical management and healthcare interactions, the latter driven by multifactorial communication difficulties. Techniques that helped included slow, simple, and single messages, avoiding topic switching. Information and emotional support needs were often unmet, particularly for caregivers. Diagnostic pathways were inconsistent and awareness of cognitive impairment in PD was poor, both contributing to underdiagnosis. Many felt that PwP and cognitive impairment fell through service gaps, resulting from disjointed, nonspecific, and underresourced services. Personalised care was advocated through tailoring to individual needs of PwP and caregivers facilitated by flexibility, time and continuity within services, and supporting self-management.

**Conclusions:**

This study highlights unmet need for people with this complex condition. Clinicians should adapt their approach and communication techniques for this population and provide tailored information and support to both PwP and caregivers. Services need to be more streamlined and collaborative, providing more time and flexibility. There is a need for wider awareness and deeper understanding of this condition and its differences from other types of dementia.

## 1. Background

Parkinson's disease (PD) is a heterogeneous neurodegenerative condition with a range of motor and nonmotor symptoms. Cognitive symptoms are common in PD and prevalence of PD dementia is 24–31%, increasing with age [1]. Cognitive impairment is associated with decreased function, lower quality of life, increased caregiver burden, and greater economic costs [2–5]. The cognitive profile differs from other pathologies: executive function and attention are typically affected earlier, visuospatial function, language, and memory later [6]. PD dementia often involves behavioral changes and aberrant perceptions [7].

The present model of care in the UK, similar to most Western countries, is that PD is typically managed under secondary care teams, led by neurologists or geriatricians, usually supported by PD nurse specialists (PDNSs), though around a third are seen by a doctor alone in clinic [8]. Therapy services and mental health services are typically available by referral though with variation in access; fully integrated teams are not common. Dementia is typically managed in the community through memory services, but the structure and provision of care vary widely [9]. The 2009 National Dementia Strategy [10], intending to include PD dementia, introduced dementia advisers and peer support networks, though not universally available. Charities also offer information and support for both PD and dementia.

Studies of related populations in PD have identified unmet needs in providing optimal care for people with late-stage PD and their caregivers; these included disjointed services, lack of continuity, and lack of flexibility [11–13]. For people with early and midstage PD and their caregivers, unmet needs include emotional support, involvement in clinical decisions, information, availability of professionals with expertise, multidisciplinary collaboration, and longer appointments [14, 15]. In the dementia field, effective, individualised, person-centered care is a key unmet need, and family caregivers describe struggling to receive support from services [16] and lack of care coordination [17]. Evaluation of the dementia advisers and peer support networks demonstrated these to help with information provision, signposting, renarration of relationships, social engagement, and empowerment [18, 19], but deficiencies remain [16, 20]. There has been growing emphasis on the role of the caregiver for people with dementia and the requirement of services to address the needs of both [16, 21–23].

Research often focuses on Alzheimer's disease or PD more generally. There is limited evidence available to guide services for those with the dual morbidity of cognitive impairment in PD. Our study's aim was to explore experiences of healthcare and support for people with cognitive impairment in PD, from the perspectives of people with PD, caregivers, and healthcare professionals. We also aimed to investigate views on how to optimise the support provided.

## 2. Methods

Qualitative semistructured interviews were used to explore experiences. Reporting is guided by the Standards for Reporting Qualitative Research framework [24].

### 2.1. Ethics

This study was given a favourable ethical opinion by the London Queen Square Research Ethics Committee (18/LO/1470) and Health Research Authority approval. All participants provided written or audio-recorded verbal informed consent.

### 2.2. Sample

Three groups of participants were purposively sampled: people with PD and cognitive impairment (PwP), family caregivers, and healthcare professionals (HCPs) working with this patient group. A range of different clinical and social backgrounds were sought for PwP and caregivers, considering age, ethnicity, education, living arrangements, duration of disease, severity of functional and cognitive impairments, geographical area, and healthcare providers. A variety of different professional backgrounds, expertise, and experience was sought for HCP participants.

### 2.3. Recruitment

Recruitment was conducted from November 2019–July 2021. Potential participants were identified and approached by clinicians in primary and secondary care or self-presented to the research team following advertisement in the charity sector. HCP participants were identified through snowballing. Potential participants were screened for eligibility (see Table 1 for criteria) and information sheets were sent via post or email.

### 2.4. Data Collection

Existing literature was reviewed to inform initial topic guide design [14, 27–30] and then refined through multidisciplinary and Patient and Public Involvement (PPI) review to ensure important issues were addressed and appropriate language was utilized. The multidisciplinary team included those with a background in geriatric medicine (JP), nursing (EC, JR), psychology (ND, JR, and MA), neurology (AS), and general practice (DN and KW). Questions covered a range of topics, with this article focussing on those relating to experiences of care and support and ideas about optimal support. Questions regarding remote consultations and experiences of living with cognitive impairment in PD have been analysed separately ([31], under review). An overview of the topic guide is provided in Supplementary File 1. Interviews were conducted by JP (clinical academic trained in qualitative research methods), in person, by telephone, or via video call, depending on COVID-19 public health restrictions and participant preference. Interviews were audio-recorded and transcribed “verbatim” with identifying information removed. Sample size was determined by information power [32], appraised throughout the data collection and preliminary analysis. The HCP group required a greater number of participants considering the broad range of disciplines involved in PD management.

### 2.5. Analysis

Transcripts were analysed using reflexive thematic analysis [33] through an inductive process. JP and EC first became familiarized with the data and then developed initial codes. These were iteratively developed through application to further transcripts (JP and EC) and wider team review. Coding was conducted in NVivo 12 software [34]. Coded data were reviewed, interpreted, and organized by shared meaning to create themes. An example of this process is provided in Supplementary File 2. Codes, themes, and supporting data were presented to the multidisciplinary team and PPI group and discussed to refine themes. This facilitated interpretation from a breadth of perspectives, giving strength to the analysis and promoting rigour through the involvement of people with lived experience of the condition.

## 3. Results

11 PwP, 10 family caregivers, and 27 HCPs were interviewed. Tables 2 and 3 show demographic details, type of cognitive impairment, and HCPs' professional backgrounds. Five interviews were conducted as PwP-caregiver dyad interviews, with one caregiver also interviewed alone. Five individual caregiver interviews were conducted where the PwP felt unable to take part. Twenty-seven were conducted by video; 13 by telephone; and four in-person. Duration ranged from 41 to 121 minutes.

**Figure 1:**
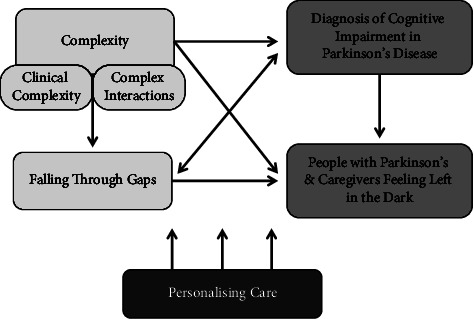
Themes, subthemes, and connections between them.

Analysis and interpretation led to five themes as illustrated in Figure 1: complexity; diagnosis of cognitive impairment in PD; PwP and caregivers feeling left in the dark; falling through gaps; and personalising care. Complexity encompasses two subthemes: clinical complexity and complex interactions. Additional sample quotes are included in Supplement 3.

### 3.1. Complexity

#### 3.1.1. Clinical Complexity

Management of cognitive impairment in PD was universally experienced as complex. Participants from all groups reported that distinguishing the cause of behaviours and symptoms could be difficult, for example, whether due to cognitive impairment or depression; motor or cognitive impairment; and cognitive impairment or medication. Assessments were complicated by fluctuations in response to medication. Condition progression and evolving complications made plans quickly outdated. Variability and unpredictability of the condition were challenging for all groups. Participants from all groups described the complex interplay of cognitive and physical aspects alongside accumulation of other medical, psychological, and social issues, often requiring input from many professionals and services. Balancing and titrating medication, often by “trial and error,” was challenging:

*“… one ailment sort of combines in with another. You try and help one symptom, but then another one goes doolally because, obviously, the medication-you know, you try and treat the Parkinson's, but then it upsets the dementia. You try and help the dementia, but then something else goes wrong.” Caregiver14*

Nonspecialist HCPs tended to defer from specialists, particularly regarding medication, but issues arose, such as basic cares being missed in the meantime and difficulty accessing specialists:

*“Because if you phone the doctor [GP], the doctor says, “Well, I don't really know what to say to you,” so. You know, “Phone up, just speak to your specialist.” Well, that's impossible.” PwP9*

HCP participants recognised that cognitive impairment in PD is common, yet several felt it neglected. Participants from all three groups perceived it to be poorly understood by the public and by other HCPs, particularly differences to other types of dementia and nonamnestic cognitive symptoms. Some advocated education and promoting awareness:

*“…people that don't work in the service don't understand, they think dementia is one thing, they don't quite understand there are lots of different dementias […] So, it's about education” HCP25 (OT, Parkinson's service)*

#### 3.1.2. Complex Interactions

Complexity also arose in healthcare interactions, reported from all three perspectives, largely due to communication difficulties arising from the combination of cognitive and physical impairments. HCPs experienced difficulties in gathering and delivering information and found conversations about progression, complex decision making, and advanced care planning, even harder in the presence of cognitive impairment. Difficulties for PwP were described by all three groups: understanding medical information, forgetting to raise issues in appointments, difficulty explaining their symptoms, struggling to understand instructions, being less motivated to follow advice, and not retaining information provided to them:

*“Even if something has been explained and spoken about, she [PwP] hasn't remembered that it's been spoken about” Caregiver15*

The caregiver's role in healthcare interactions increased with progression of cognitive impairment, with associated challenges for all involved. Despite perceived necessity or in some cases it being requested by the PwP, this caused dissatisfaction, concerns about exclusion of the PwP, pressure on the caregiver, and potential for conflicting opinions:

*“I haven't found a satisfactory way other than, as much as I hate to admit it, side-lining the patient and talking to carer and making decisions with the carer.” HCP14 (Neurologist)*

Recommendations to improve interactions were made from participants across the groups, as detailed in Table 4, including adapting the clinical approach, communication techniques, and strategies for balancing PwP and caregiver voices. However, many also cautioned about patronising:

*“not to complicate the conversation, just talk about one thing at a time, not in a patronising way but just not to bring in other threads of conversation” HCP23 (Geriatrician)*

### 3.2. Diagnosis of Cognitive Impairment in PD

A range of participants from all groups described PwP not being given a clear cognitive diagnosis. Variation in diagnostic pathways was evident. The poor understanding of cognitive impairment in PD described above also impacted identification of symptoms. Fewer than half of the PwP represented had accessed memory services. Participants from across the groups reported access issues such as long waiting times; several caregivers perceived the requirement to go via the GP as an unnecessary hurdle in the pathway to memory services:

*“the neurologist has been asking for my mother to have a geriatric memory test and I can't get through the GP to get that done. There's quite a long waiting list for it.” Caregiver10*

Some neurologists and geriatricians preferred to diagnose cognitive impairment in their own service, not perceiving additional value from memory services. Conversely, diagnoses made outside of memory services were not always explicit:

*“I think one of the biggest things is the fact that these people are under lots of different services. And what I've found before is that sometimes neurologists may start medication for dementia without telling the person that they have dementia.” HCP2 (Psychiatrist)*

*“When he's been to the Parkinson's centre, he's occasionally been assessed for cognitive ability… he actually quite clearly showed, quite a big deficit in things like mental arithmetic and anything noticing patterns, visual patterns I think. I can't remember everything they did. Remembering words… So I think it has been established, he's got some score on some piece of paper somewhere [laugh]… I'd be interested in hearing about a memory clinic.” Caregiver2*

Some PwP desired clarity regarding their cognitive diagnosis, others did not wish to “*open up that can of worms*” (PwP13).

*“No, I haven't and I haven't even discussed that [cognitive changes] with [wife], because that could lead onto other things and I really don't want to think about that.” PwP5*

Many HCPs and several caregivers expressed that formal diagnosis was important for optimal support and treatment:

*“[addressing cognitive impairment] can have very important implications in terms of the care we can provide them with, the support we can provide them, and even with the medication we may choose for them in the future” HCP16 (PDNS)*

However, the two directly interviewed PwP who had received dementia diagnoses (note one was recent and would not use the word “dementia”) still felt unclear about what it meant for them:

*[Having talked to their HCP about cognition-] “I didn't get any much-any more information than I had before.” PwP15*

*[Asked if wanted to know more-] “In one way no, but I've got to be sensible because it's really after me, it's [spouse], has got to sort himself out as well. So yeah, I would like to know more.” PwP3*

Receiving a diagnosis could be burdensome. Misunderstanding of cognitive impairment in PD could lead to feelings of “*blame or shame*” (HCP9, Psychologist) and potential discrimination within healthcare:

*“I think if someone has got dementia they're taken less seriously.” HCP1 (Psychiatrist)*

Many participants were unsure what the ideal pathway would be, but several depicted a need to define standardised pathways:

*“clear streamlined pathways that are consistent across, so everyone with Parkinson's has the same level of care.” HCP9 (Psychologist)*

Many expressed that the key was a collaborative and holistic approach, encompassing physical, cognitive, psychological, and social aspects. Some HCPs felt there was a need to normalise conversations about cognition to achieve this.

### 3.3. PwP and Caregivers Feeling Left in the Dark

Participants across the groups were concerned PwP with cognitive impairment and caregivers were inadequately informed. Amount and type of information desired were largely personal preference, and many had mixed feelings. Typically, caregivers wanted more information than PwP, particularly personalised feedback to manage expectations and enable planning, whereas PwP desired advice for the present. A few participants described information overload, but most depicted a deficiency:

*“You're going in the dark really all the time and having to change and adapt […] I don't think anything's set out and explained very well anywhere.” Caregiver6*

Challenges also arose from difficulties accessing and understanding information provided, exacerbated by cognitive symptoms or caregiver stress. Within extensive critique of information resources, several emphasised the lack of specific and relevant information:

*“…with Parkinson's, it's always the generalised aspects of it, the shaking, with the tremor, with Parkinson's, and my mum doesn't even have that. For dementia, it's memory more, and with my mum, I would say it's more hallucinations, the delusions, the-so the unawareness, the confusion is more of a big deal.” Caregiver15*

Participants also reported feeling unsupported in aspects beyond physical needs. Some caregivers and some HCPs, typically from mental health services, perceived that HCPs from other health settings sometimes neglected psychological and social needs. Although medications were depicted as important, some from each group felt that doctors can be too medication-focussed:

*“it would be nice if there was…a better response to when you say “this is happening” than just, “Oh, we'll up the drugs.” If they could explain it better, I think I'd feel happier” Caregiver10*

Consideration of psychosocial wellbeing by all HCPs involved was advocated:

*“psychological care is everybody's business” (HCP9 Psychologist)*

Furthermore, a need for focussed and responsive emotional support was also expressed:

*“…if you've got a problem comes up and you need some reassurance. But I think you need someone to be able to say, “Can you help me out?” You know, “I”m dealing with so-and-so and I can't cope with it.” PwP9*

In exploring potential provision of emotional support, personal qualities and skills appeared more important than specialist knowledge, and for some, peer support could provide it:

*“Sharing sensible reflections on these things is useful. And I think it helps in a way in terms of kind of mental pressures of dealing with Parkinson's” PwP1*

It was apparent that many caregiver participants, particularly those caring for relatives with PD dementia, were struggling or felt “*desperate*” (Caregiver14).

Positive experiences of information and support from all three groups commonly came from the charity sector, particularly for advice on practical and financial issues such as benefits and respite and for groups and activities. “Drop-in” models reportedly worked well, as did combined PwP-caregiver support. PDNSs were widely considered an important support, when available and accessible.

### 3.4. Falling through Gaps

It was apparent from all three groups that current services do not meet the needs of this population. This was perceived to be because services were disjointed; not designed for cognitive impairment in PD; and due to lack of capacity. The clinical complexity demands input from a range of services, increasing with disease progression, and participants universally felt that more time was required for care of this population.

All three groups reported difficulties in navigating pathways and systems. HCPs experienced a lack of communication and information sharing between teams and some described uncertainties over their roles and “*lines of accountability*” (HCP21, Psychiatrist). This existed between mental health and PD teams; primary and secondary care; and health and social care, exacerbated by regional variation and frequent reconfiguration of services:

*“…there's not enough connection between acute trusts and mental health trusts. And the two will very often not communicate at all, which is not at all helpful for the individual stuck in the middle.” HCP27 (Physiotherapist)*

HCPs felt this compromised their care delivery and hindered appropriate signposting or referral.

This was consistent with the experiences of many PwP and caregivers who were frustrated by lack of connection between services and not knowing what is available. Many caregivers described a consuming search, going “*round in circles*” (Caregiver14) to access services or getting caught up in “*a lot of bureaucracy*” (Caregiver7). Services required effort to “*chase*,” without which people reached “*crisis*” before receiving help. Cognitive impairment was considered a barrier to help-seeking and there was heavy reliance on proactivity of caregivers, so significant concern raised for those without caregivers:

*“…those people who don't have anyone can have difficulties accessing that because of their memories.” HCP3 (Dementia Nurse Specialist)*

Like for diagnosis, opinions were mixed about the role of memory services for postdiagnostic care in PD. Many HCPs felt one-off review, as provided by most memory services, was unsuitable for PD dementia, and similarly, several caregivers expressed disappointment at the lack of long-term input. Memory services were said to be designed for other types of dementia, limiting their usefulness in PD:

*“part of the difficulty with that is they're very much an Alzheimer's or mixed dementia, whatever that is, service. So it's unfortunately not a tailored Parkinson's dementia service.” HCP11 (Neurologist)*

On the other hand, some HCPs felt the memory service approach was more holistic and better positioned to signpost other services:

*“we've, kind of, linked in with the third sector and much more local resources which the tertiary services, or even the neurologist, may not be aware of.” HCP21 (Psychiatrist)*

Similarly, one caregiver spoke highly of a dementia charity support, referred to by the memory service. Some HCPs reflected that they did not signpost to dementia charities due to perceived lower value in PD, despite thinking the support may help.

Participants from all three groups raised concerns about insufficient capacity. Limited time and frequency of Parkinson's appointments generated pressure:

*“It's a real difficult appointment, obviously, because you know if you don't get it right in that 10–15 minutes, it's going to be a year before you'll be able to pick up on it again.” Caregiver10*

HCPs felt unable to deliver optimal care within capacity constraints, for example, being unable to offer “home visits,” or unable to fully discuss complex decisions:

*“I think it [complex decisions] involves having the conversations on a number of occasions and just don't have the resources.” HCP14 (Neurologist)*

This was exacerbated by limited access to input between appointments:

*“I mean, you can leave 100 messages and you never hear.” PwP9*

Mental health input was limited. Carer support provision was widely considered “*so lacking*” (HCP22 PDNS). Moreover, many perceived injustice from the “*postcode lottery*” (multiple participants) of service variation.

Navigation and capacity issues were longstanding, but all found them exacerbated by the COVID-19 pandemic. Services and groups were being suspended or changed to remote delivery, adding uncertainty about service availability. HCPs reported added pressures and disconnection relating to staff redeployment, changed ways of working and diminished teams, and acknowledged by several PwP and caregivers.

Despite the widespread reported problems, some positive experiences were described. Some HCPs described advantages of their multidisciplinary teams utilising different skillsets to collectively be holistic and learning from each other. Most HCPs desired or were seeking more collaborative working building professional relationships and integrating services. At the simplest level, this involved sharing contact details and informal communication between teams; a step up involved joint reviews; and requiring the most change fully integrating services. The few HCPs that worked in integrated services, with a multidisciplinary PD team and psychiatrist working side-by-side, perceived them to be more efficient with higher quality-of-care. Central to suggested solutions from all groups was information sharing between services (e.g., management recommendations and conversations held), ideally with integrated electronic records:

*“ideal world […] seamless communications with computer systems which talk to each other and people who talk to each other.” HCP23 (Geriatrician)*

Notwithstanding the negative consequences of COVID-19, several HCPs commented that the implementation of remote technology during the pandemic facilitated more efficient multidisciplinary meetings and reviews.

To improve service navigation, participants sought clarity of roles and responsibilities of different health and social care providers. Proactive bespoke signposting was proposed to improve access. Some participants from all groups recommended a “single point of contact” or a “*dedicated line*” (Caregiver7). Most felt this should reside within the PD specialist team but coordinate with others:

*“… just one contact that could then contact everyone else that is involved in that person's care.” HCP2 (Psychiatrist)*

“Out-of-hours” times were a challenge, with issues often arising at night, so some proposed a 24-hour support line.

“Ideal world” descriptions tended to involve increasing resource and capacity. Many emphasised that services would be better if targeted specifically to PwP and cognitive impairment. Increased flexibility was desired, to allow appointments at best time of day for the individual, for optimal medication effect, and to be responsive to changing needs. Most expressed that duration of appointments needed to be longer for this population, but one proposed short appointments more frequently to reduce tiring and facilitate reinforcement.

### 3.5. Personalising Care

Personalised care was perceived to be founded on strong relationships among PwP, caregiver, and HCP, more achievable with continuity of care provider. It was hindered by insufficient time and PwP feeling rushed or burdensome. Better encounters were described when HCPs displayed good knowledge, engagement, and attentive listening:

*“[doctor] always took time with him and everything, to listen and talk about his Parkinson's” Caregiver4*

*Explaining a positive encounter with an OT: “Because they came and sat down near me and talked to me.” PwP11*

To personalise care, participants from all three groups described how HCPs should identify what the PwP and caregiver want to know and how they want support, without making assumptions, considering a range of factors as listed in Table 5:

*“I think, try and find out what they want to know. Try and find out what they're scared of. Don't just make assumptions; don't just bombard them with lots of information that they don't know they need to know. Try and gauge, tailor, what you can give them by what they-you can … You need to be able to perceive what they need, but you need to ask people. “What would you find helpful?” “What do you need to know?” […] It's not a one-off thing; it's a process.” Caregiver12*

Recognising different needs of PwP and caregivers appeared important to maintain strength in the care partnership:

*“information needs to match emotionally what people feel they're able to cope and manage and hear. But it also needs to match cognitively … [caregivers] need information in a different way and that's OK, let's meet their needs as well as the patient's…” HCP9 Psychologist*

To deal with individual differences in processing information, the ideal scenario described by one HCP involved an assessment of the individual that would be shared across services:

*“…if somebody was able to assess an individual and work with them to find out what is the best method of receiving information, and then that is conveyed to all the professionals and all the voluntary sector. And they can say to someone, “this is the best way for me to get information,” rather than us all kind of scrabbling around and just being a bit lost and feeling inadequate” HCP6 (SLT)*

This was mirrored by caregiver and PwP desire for their personal situation to be better shared and understood:

*“I think one of the things is like back to treating him like an idiot. It'd be really good if they could understand what he … If there was some way of letting professionals know where he was. So at this stage if you wait, [PwP] will be able to answer. […] you do need the professionals. It's vital that you have a relationship with them, but it could somehow be communicated the stage you were at.” Caregiver11*

Some participants from each group expressed interest in self-management:

*“Rather than just hear I'm finding it more difficult, which is probably true, I'd like to have a session on how to get better or how to deal with them better or how to do mental exercises every day, I'd quite happily do them.” PwP1*

Cognitive impairment was sometimes seen as a barrier, particularly by HCPs. Nondoctor HCPs appeared more confident in adapting recommendations for self-management to cognitive impairment by maintaining familiarity (e.g., environment or equipment), and maximising assets, “*trying to work around what they've actually got at the moment*” (HCP12, PUK Adviser), rather than introducing new concepts. Many reported the value of physical activity, social interaction, and engaging cognitive functions, although only one PwP had undertaken formal cognitive stimulation therapy. For severe cognitive impairment, HCPs directed advice to caregivers; however, many warned of deskilling the PwP. Instead, confident HCPs advocated caregiver education to encourage independence: prompting; simplifying tasks; breaking down instructions; focussing on positives and solutions whilst allowing mistakes:

*“…helping family members or carers to adapt their input, which allows that functioning to happen, to maximise somebody's potential rather than taking skills away from people.” HCP5 (OT, Memory Service)*

## 4. Discussion

### 4.1. Summary of Findings

PwP, caregivers, and HCPs all considered cognitive impairment in PD to be complex: the myriad of interacting symptoms and management complexities, alongside difficult healthcare interactions resulting from multifactorial communication problems and balancing PwP and caregiver involvement. Unclear diagnostic pathways plus poor awareness of cognitive symptoms in PD contributed to underdiagnosis. Many PwP and caregivers felt “left in the dark” due to lack of suitable information and emotional support. Services were experienced as disjointed, nonspecific, and underresourced. As shown in Figure 1, complexity fed into these challenges around diagnosis, support and services, and underdiagnosis was perceived to be a barrier to accessing services and receiving support. Improvements were proposed: clarity of care provider roles, information sharing, service integration, and better signposting. Services need increased flexibility, time, and continuity to serve this population. Personalised care requires exploring and tailoring to individual needs of both PwP and caregivers, building on established relationships; and supporting self-management.

### 4.2. Context of Existing Literature

The complexity of PD and the delicate balancing act for pharmacological management in presence of cognitive impairment is recognised [36]. Our study offers further insight, highlighting the real-life challenges of providing care to those with this complex condition. Consistent with previous reports [11, 36, 37], identifying the cause of symptoms could be difficult and fluctuations complicated assessment and management, emphasising a need for those providing care to understand the nuances of this condition. Communication impairments are known to be multifactorial in PD [38], so difficulties in healthcare interactions are not surprising, but the inclusion of the three participant groups in the present study showed how challenging these were from all three perspectives and were almost universal. Communication impairments have been associated with worse outcomes in acute care [39] and dissatisfaction with medical care [40] so are really important to address. Similar to our findings, the role of the “partner” has been reported to be both a facilitator and barrier to consultations by PDNSs [41], and risk of exclusion of the patient has been reported in dementia research [42]. Techniques described by our participants corroborate and expand on those reported by PDNSs [41], particularly through inclusion of PwP and caregiver experiences. These additional perspectives are invaluable for developing recommendations to avoid a paternalistic approach and highlighting potential different interpretation of encounters, for example, a caregiver cautioned against checking comprehension of the PwP with caregivers, since it can appear patronizing.

Different diagnostic pathways were described by participants from different regions, and diagnoses were often been missed. There were mixed views regarding obtaining a cognitive formal diagnosis, with many PwP being hesitant about investigating symptoms, but diagnosis was broadly perceived as valuable by HCPs and caregivers, consistent with broader dementia research [43, 44]. Evidence suggests that the value relies of obtaining a diagnosis rests in it being accompanied by information and support [45–47] and unfortunately the participants who had received dementia diagnoses appeared to have lacked this (though one was only recent). Some participants positively described a holistic approach from memory services; others (typically from PD services) did not feel memory services added value in PD. At the heart of this was the perception that existing services and resources were designed for other types of dementia and not suitable for this population, e.g., lack of long-term follow-up. Only one PwP (represented by their caregiver) had accessed cognitive stimulation therapy despite evidence of benefit in dementia on the whole [48] (a range of dementia pathologies including PD-dementia were included) and it being in clinical guidelines [49]. This is perhaps due to the variation in pathways accessed by PwP, and cognitive stimulation therapy is typically delivered via memory services. Improving postdiagnostic care for PD-dementia could enhance the value of the diagnosis, and streamlining the diagnostic pathway could improve rates of diagnosis and access to available support.

Information and emotional support were considered deficient by many participants, particularly for caregivers. The relational context for care and sustaining relationships has been called “essential” for welfare of people with dementia [50] and the desire for treatment as a care team is apparent [16, 21–23]. Many participants of the present study from all participant groups advocated addressing needs of both PwP and caregiver as normal practice, though many HCPs found this difficult in practice typically due to capacity constraints. As for our participants, fragmented services and access issues have challenged both dementia care [51–53] and PD across the stages [22, 54]. However, these issues were most prominent for PwP with more severe impairments and their caregivers in our study. This is unsurprising since cognitive impairment can be a barrier to accessing healthcare in PD [55], and our participants described cognitive impairment exacerbating navigation difficulties and processing of information. Interestingly, the inclusion of HCPs in the present study revealed that these difficulties and frustrations are not limited to service users but applied to HCPs too, with structural issues perceived to prevent optimal care delivery.

There is evidence for integrated palliative care compared to standard care [56] and integrated multidisciplinary team working [57, 58] for services for PwP and other long term conditions. In the present study, interdisciplinary collaboration was considered especially important by all three participant groups in the context of dual physical and cognitive impairment. As with past studies across PD stages [11, 14], support was not always person-centered. Although the literature is sparse, person-centered care models are associated with improved management (adherence to quality of care indicators) [59] and improved symptoms and quality of life [60] compared to standard care in PD. The former study utilised nurse care managers to coordinate care and develop action plans with the PwP that included problem-specific interventions such as information, problem-solving collaboratively, and clinical referrals. The latter included the development of a treatment plan, which was individualised and dynamic with regular home visits from a PD nurse and a telephone hotline. These studies excluded participants with PD-dementia, but the approaches taken fulfil many of the desires of the participants of the present study so further exploration in this population would be valuable.

Furthermore, facilitators of personalised care described by our participants are consistent with important factors for care delivery described in the literature, such as established relationships and knowledge of the condition [41, 61]. Studies of self-management in PD have often excluded those with cognitive impairment [62] but HCPs in the present study described tailoring it for cognitive impairment. This involved working with what the person has, maintaining familiarity, and teaching caregivers to support the PwP whilst avoiding deskilling, for example, breaking down tasks rather than completing the task for them. This resonates with the concepts of “co-operative communication,” “co-operative action,” and “co-operative care” identified with people with dementia and their caregivers, promoting a sense of solidarity [50]. Future studies of self-management in PD should incorporate this into the intervention and include participants with cognitive decline.

### 4.3. Key Challenges

Our study highlights important areas of incongruity within care provision for people with PD and cognitive impairment. Wider understanding and awareness of cognitive impairment in PD was perceived to be poor, management and interactions complex, and nonspecialist HCPs deferred to specialists; yet access to specialists was problematic and services were not specific or suitable for cognitive impairment in PD. Multifactorial communication impairments impede healthcare encounters, and techniques to manage this involve a slower pace and more time; yet there is insufficient time in appointments to employ these. Inconsistencies in pathways meant cognitive diagnoses were often not formally made; yet some support services were perceived to be exclusively available to people with dementia diagnoses. People with PD and caregivers felt uninformed and unsupported; yet available services were difficult to access and navigate, even by HCPs. Reliance on caregivers was high; yet caregiver support was lacking and people without caregivers were considered especially vulnerable.

### 4.4. Clinical Implications

At an individual level, it is essential that clinicians develop communication skills to manage these challenging healthcare interactions, which could utilise our participants' recommendations in Table 4. Awareness of cognitive impairment in PD and education about the nuances of the condition needs to be more widely promoted to enable better understanding by all health and social care providers. Clinical practice could be improved through personalisation of care tailoring to individual needs of PwP and caregiver and supporting self-management. To do this, clinicians need to utilise clinical knowledge, establish relationships, and listen attentively, identifying individual needs and recognising differences, considering factors detailed in Table 5, and ensuring this is a dynamic process rather than a one-off matter.

### 4.5. Service Implications

At a service level, more time needs to be provided for these complex cases, to give clinicians the time to employ the required communication techniques and personalisation of care whilst ensuring both PwP and caregiver needs are addressed. Services should promote continuity of care. Models of care that facilitate specialist input when needed should be developed. Ideally, this would involve radical change to develop a responsive and accessible specialist service, including telephone line and home visits, and outreach to those without caregivers, but since capacity and resource constraints are unavoidable, optimising existing services may be more realistic. Optimisation would involve record-sharing and communication networks, for example, between primary and secondary care and between PD and memory services. To improve navigation, cross-service collaboration is necessary: clear roles need to be defined and mechanisms of contact established and then these must be made known to both care providers and service users.

Policies and services typically use the umbrella term “dementia” but do not discriminate between types of dementia [10, 18], yet our study highlights that the differences are important. Information resources and support services designed specifically for this population would likely be beneficial. Whilst PD dementia is not the most common dementia, PD prevalence is increasing [63] and cognitive impairment increases with age and duration of PD [1, 64], so investment in services now may help prevent greater numbers of people feeling “alone in the dark” in future.

### 4.6. Research Implications

Our study highlights the need for research specifically addressing cognitive impairment in PD, rather than grouping all dementia pathologies under one umbrella. Since cognitive impairment and dementia have frequently been exclusions in PD studies, it also invites further investigation of self-management and models of care for this population.

### 4.7. Strengths and Limitations

A range of individual demographic factors and professional backgrounds are represented. Conducting the study remotely enabled wider geographical coverage and so perspectives from a range of health services. The use of the three groups of participants enables consideration of healthcare from the perspectives of both service users and service providers, affording increased depth of understanding. Whilst the inclusion of a greater number of HCPs compared to PwP and caregivers was intentional, to represent the range of disciplines involved in PD care, it potentially makes the HCP voice more dominant. Awareness of this was however maintained throughout the analysis. Inclusion of participants with subjective cognitive symptoms rather than formal diagnosis prevented being restricted by underdiagnosis, a recognised problem [25]. Conversely however, we cannot interpret our findings within the context of objective severity of impairment. The multidisciplinary research team and PPI helped interpretation. An unavoidable challenge for research with this population is participant communication difficulties. Some had difficulty expressing their views and caregivers' proxy views could be biased. Whilst the range of professional backgrounds represented brings richness to this data, regional variation in health services must be recognised: many PwP will not routinely encounter this range of specialist professionals [8]. All PwP participants were under the care of a specialist, though the input varied. Those entirely disconnected from specialist care were not represented, which may warrant further investigation.

## 5. Conclusion

This study furthers our understanding of the complex needs of PwP with cognitive impairment, reinforcing the need for a holistic, tailoring approach to care, and addressing needs of PwP and caregivers. The findings illustrate a need for streamlined diagnostic and care pathways, increased resource and flexibility to address the complex needs in the presence of the dual challenges, interdisciplinary collaboration, and service integration, with clearer signposting and communication. The differences between cognitive impairment in PD and other types of dementia need to be reflected at the individual level, service level, and within research, with awareness promoted more widely.

## Figures and Tables

**Table 1:** Inclusion and exclusion criteria.

Samples	Criteria
People with Parkinson's disease and cognitive impairment (PwP)	(1) Diagnosis of Parkinson's disease made by a clinical specialist
	(2) Cognitive impairment identified by clinician and symptoms recognized by participant^*∗*^: described in lay terms as “changes in memory, thinking, and concentration”
	Exclusions: care home residents, atypical Parkinsonian disorders, participants anticipated to be approaching end of life

Family caregivers	A family member or friend who closely supported the person with PD, whether or not they identified as a “carer”
	Person being supported needed to meet the abovementioned inclusion criteria

Healthcare professionals (HCPs)	A person working within, or in collaboration with, healthcare, who encounters people with PD and cognitive impairment in a professional capacity

^*∗*^Participants reporting subjective cognitive symptoms, having been identified by a clinician as having cognitive impairment, were included even in the absence of formal diagnosis since cognitive symptoms are often underdiagnosed in clinical practice [25, 26]. Participants were not included if they denied cognitive symptoms despite a clinician identifying them, since it would not be appropriate to attempt detailed interview discussion of these symptoms with them.

**Table 2:** Demographic details for people with Parkinson's disease and caregivers.

People with Parkinson's represented		

	Interviewed directly (*n* = 11)	Represented by their caregiver (*n* = 4)

Cognition	8 reported subjective symptoms (varying severity)	4 formal diagnoses of dementia
	1 formal diagnosis of mild cognitive impairment	
	2 formal diagnoses of dementia	

Age	Mean 73.7 yrs (SD 6.9)	Mean 81.0 yrs (SD 10.2)
	Range 59−86 yrs	Range 74−96 yrs

Sex	8 males	1 male
	3 females	3 females

Ethnicity	9 White (British)	1 Asian (Indian)
	1 White (others)	3 White (British)
	1 Black (others)	

Duration of PD	Mean 13.2 yrs (SD 7.1)	Mean 14.5 yrs (SD 7.1)
	Range 2–25 yrs	Range 8−20 yrs

Schwab and England scale^*∗*^	Mean 57.5% (SD 28.2)	Mean 22.5% (SD 18.9)
	Range 10–100%	Range 10–50%

Living arrangements	5 living with spouse/partner	1 living with spouse
	3 living with family	1 living with family
	3 living alone	2 living alone

Educational background	Age leaving full time education ranged from 14 to 25 yrs	
	Qualifications range from none, through to degrees	

Location	13 urban/suburban	
	1 semirural	
	1 rural	
	All from the southeast or east of England	

Caregiver participants		

	Caregivers of participating people with Parkinson's (*n* = 6)	Caregivers where person with Parkinson's did not (*n* = 4)

Relationship	3 spouse	2 spouse
	3 daughters	2 daughters

Age	Mean 60.2 yrs (SD 9.9)	Mean 66.8 yrs (SD 13.1)
	Range 46−70 yrs	Range 48−78 yrs

Sex	2 males	1 male
	4 females	3 females

Ethnicity	5 White (British)	3 White (British)
	1 Black (others)	1 Asian (Indian)

Location	All from the southeast or east of England	

^*∗*^indicates degree of dependence, with 100% being independent and 0% being fully dependent [35].

**Table 3:** Backgrounds of healthcare professional participants.

Professionals (*n* = 27)	
From the southeast of England, the Midlands, and Scotland	
6 specialist nurses	4 Parkinson's disease nurse specialists (PDNS)
	2 dementia nurse specialists

13 doctors	3 neurologists
	3 geriatricians
	3 psychiatrists
	3 general practitioners (GP)
	1 palliative care physician

7 Allied health professionals	2 clinical psychologists (neurological services)
	2 occupational therapist (OT)-1 Parkinson's services, 1 memory service
	2 speech and language therapists (SLT)-neurological services
	1 physiotherapist (Parkinson's service)

1 charity sector	1 Parkinson's UK local adviser^*∗*^

^*∗*^Charity sector role to help PwP, including providing advice and information and supporting access to services.

**Table 4:** Participant recommendations to improve interactions.

Clinical approaches	Communication techniques	PwP-caregiver dynamic
(i) Select the priority issues to address	(i) Consider use of images	(i) Allocating caregivers specific time to discuss their concerns about the PwP and also to explore their own needs
(ii) Make a single recommendation at any one time	(ii) Consider simple hand signals to supplement verbal communication (e.g., touch the chair when asking someone to sit)	(ii) Simultaneous multiprofessional consultations: PwP and caregiver each with a different HCP
(iii) Regularly reinforce recommended strategies	(iii) Speak slowly and simply, with concise phrases	
(iv) Minimise anxiety, e.g., calm environment; utilise familiarity (e.g., could lead the conversation from photographs); use of music; if a topic provokes anxiety, pause and come back to it later	(iv) Allow time, use pauses, listen patiently	
(v) Be flexible, e.g., optimal timing for the PwP; let pace be guided by the PwP, recognise what aspect of the interaction is difficult for the PwP; and split discussion into shorter conversations if needed	(v) Reflect back and check understanding with the PwP (caution: asking the caregiver about PwP understanding may be perceived as patronising)	
(vi) Be open about what to expect	(vi) Be mindful about language (e.g., the term “dementia”)	
(vii) Normalise advanced care planning topics	(vii) Directly acknowledge communication difficulties, reassure and ask how the PwP wants them to be handled	
	(viii) Minimise topic switching	
	(ix) Explain purpose before launching into questions	
	(x) Prompt, without dwelling on the forgetfulness	
	(xi) Utilise techniques identified as helpful by others involved (e.g., SLT recommendations)	

See Supplementary File 3 for sample supportive quotes.

**Table 5:** Factors identified by participants to consider when tailoring support.

Personal factors	Condition-related factors
(i) Preferences, e.g., for written or verbal information; what they want to know; and personal priorities	(i) Degree of cognitive impairment
(ii) Personality	(ii) Particular challenges and symptoms
(iii) Level of proactivity and motivation	(iii) What is going well (“assets”)
(iv) Personal interests	(iv) Expected benefits of the recommendation for that individual
(v) Language	(v) Practical barriers, e.g., mobility and transport use
(vi) Culture	
(vii) Reading ability	
(viii) Educational background	
(ix) Others involved in care	

See Supplementary File 3 for sample supportive quotes.

## Data Availability

All supporting data are included in the article and supplementary materials. The full transcripts are not available publicly due to ethical requirements.

## Additional Points

*Key Points*. (1) Cognitive impairment in Parkinson's leads to complex needs and often heavy reliance on caregivers' support and initiative. (2) Services and information resources are not currently well suited to the complexity of the dual challenges in the condition. (3) Services should be more accessible, joined-up with accessible records, and flexible to cater to the needs of this population. (4) Clinical approaches should be adapted for cognitive impairment and support tailored to individual patient and caregiver needs. (5) Greater awareness and understanding of nuances of this condition are needed, recognising the differences from other dementias.

## Supplementary Materials

Supplementary Materials Supplementary File 1: Overview of the topic guide for interviews. Supplementary File 2: Example of analysis process. Supplementary File 3: Additional participant quotes.
