# Supplementary material for: Delivering Optimal Care to People with Cognitive Impairment in Parkinson's Disease: A Qualitative Study of Patient, Caregiver, and Professional Perspectives
Source: Parkinsons Dis. 2023 Aug 29;2023:9732217. doi: 10.1155/2023/9732217 (PMC10480026; doi:10.1155/2023/9732217)
Supplement: Supplementary Materials — Supplementary File 1: Overview of the topic guide for interviews. Supplementary File 2: Example of analysis process. Supplementary File 3: Additional participant quotes. [file 9732217.f1.zip › Supplement 1 - Topic Guide Overview.docx]

*Note language was adapted to the individual interviewee for sensitivity. Where dyadic interviews took place the guides for people with Parkinson’s and caregivers were blended to maintain flow of discussion whilst ensuring both voices were heard.*

**Interview Guide for People with Parkinson’s**

1. **Condition and management**

*Parkinson’s in general, and cognitive impairment:*

- Overall experience & perception
- Difficulties
- Current management
- Facilitators
- Barriers
- Others consulted
- Changes over time

1. **Current help**

- Sources and experience of support
- *Parkinson’s in general, and cognitive impairment*
- *Personal, professional, charity*
- *People, services & information resources*
- *Parkinson’s nurse*
- *Memory clinic/service*
- Most & least helpful
- Unmet need
- What optimal support would be

1. **Experience and views of remote support**

- Telephone
- Internet/online
- Healthcare

**Interview Guide for Caregivers**

1. **Current situation**

- Role
- Difficulties/challenges
- *Parkinson’s in general, and cognitive impairment*
- Barriers to optimal management

1. **Maintaining wellbeing and independence**

- Facilitators for person with Parkinson’s
- Facilitators for caregiver

1. **View on help, information and resources**

- What do you need support/help with or information about?
- *Parkinson’s in general, and cognitive impairment*
- Experience of existing information/resources
- Impact of caring & support needed
- What optimal support would be

1. **Experience and views of remote support**

- Telephone
- Internet/online
- Healthcare

**Interview Guide for Healthcare Professionals**

1. **Role & Experience**
2. **Difficulties in management**

(Parkinson’s + cognitive impairment)

1. **Reports from people with Parkinson’s and caregivers**

*If not covered – involvement of Parkinson’s nurse? Involvement of memory services?*

1. **Where do you recommend for information & support?**
2. **How support could be best provided**

*If not covered – role of Parkinson’s nurse? Role of memory services?*

1. **Remote consultations**
