# Supplementary material for: Delivering Optimal Care to People with Cognitive Impairment in Parkinson's Disease: A Qualitative Study of Patient, Caregiver, and Professional Perspectives
Source: Parkinsons Dis. 2023 Aug 29;2023:9732217. doi: 10.1155/2023/9732217 (PMC10480026; doi:10.1155/2023/9732217)
Supplement: Supplementary Materials — Supplementary File 1: Overview of the topic guide for interviews. Supplementary File 2: Example of analysis process. Supplementary File 3: Additional participant quotes. [file 9732217.f1.zip › Supplement 2 - Example Analysis.docx]

**Themes**

Falling Through Gaps

People with Parkinson’s & Caregivers Feeling Left in the Dark

- Carer Support
- Joining up & navigating care
- Lack of HCP time or resource
- MDT & professional teamwork
- Responsiveness of healthcare
- Service Structures
- Sharing Records
- Suboptimal Healthcare Interactions

*Services not meeting the needs of this group – capacity, non-specific and structural organisation*

- Carer or Family Challenges
- Critique of information resources
- Critique of Support Services
- Peer Support
- Sources of Information & support

*PwP and Caregivers feeling inadequately informed and unsupported.*

- Carer or Family Challenges
- Carer Support
- Critique of information resources
- Critique of Support Services
- Discussing the Future
- Joining up & navigating care
- Lack of HCP time or resource
- MDT & professional teamwork
- Peer Support
- Responsiveness of healthcare
- Service Structures
- Sharing Records
- Sources of Information & support
- Suboptimal Healthcare Interactions
- Carer or Family Challenges
- Critique of information & support
- Joining up & navigating care
- Lack of HCP time
- Local services
- Sources of Information & support
- Suboptimal Healthcare Interactions

**Grouping Codes & Looking for Meaning**

**Revised Codes (Sample)**

**Initial Provisional Codes (Sample)**
