# Supplementary material for: Delivering Optimal Care to People with Cognitive Impairment in Parkinson's Disease: A Qualitative Study of Patient, Caregiver, and Professional Perspectives
Source: Parkinsons Dis. 2023 Aug 29;2023:9732217. doi: 10.1155/2023/9732217 (PMC10480026; doi:10.1155/2023/9732217)
Supplement: Supplementary Materials — Supplementary File 1: Overview of the topic guide for interviews. Supplementary File 2: Example of analysis process. Supplementary File 3: Additional participant quotes. [file 9732217.f1.zip › Supplement 3 - Additional Supportive Quotes.docx]

| **Theme** | **Sample Quotes** |
| --- | --- |
| **Complexity**  Subtheme: Clinical complexity | “I think it’s the hardest group of people to work with full stop” HCP21 (Psychiatrist)  “if it’s not the Parkinson’s, it’s the medication, and the medication could be more of a problem than the Parkinson’s” C15  “So often if we’re referred someone with Parkinson’s and they’ve got psychiatric problems -they might be hallucinating - actually it’s because of their Parkinson’s drugs and if they’re on dopaminergic drugs. And then of course I’m very cautious about messing around with drugs without getting hold of some neuro advice.” HCP1 (Psychiatrist)  “as soon as somebody comes in on an acute take, the first thing is, ‘Get a Parkinson’s review.’ And actually, some of the aspects of good general care can be forgotten.” HCP10 (Geriatrician)  “I often find personally it’s sometimes difficult whether to know whether they're being under treated or over treated at times when it comes to the Parkinson’s, because obviously you can get the hallucinations and a number of the cognitive issues obviously in Parkinson’s dementia, but you can also get that in side effects of the Parkinson’s medications. So yes, I often liaise with the Parkinson’s team quite a lot about that” HCP13 (GP)  “And even though I’ve seen many patients over the years who have Parkinson’s, I’m not really an expert. I’d love to be able to have a formal kind of training… maybe if mental health nurses or dementia specialists had appropriate training, then that would help overall. Because even if they were to start increasing the numbers of Parkinson’s nurses, the reality is there’s never going to be enough.” HCP7 (Nurse, Memory Service) |
| **Complexity**  Subtheme: Complex Interactions | “… often because of the ‘white coat syndrome’ you don’t really remember things you would otherwise want to raise.” P1  “majority of the time I have to rely on the information from the relatives and carers. Because what the patient reports is not necessarily… reliable. […] Majority of the time it is through the carer and I feel guilty of that.” HCP4 (PDNS)  “… [quoting self to PwP] “do you want to speak?” [quoting PwP:] “Uh, you just tell them [doctor]. Just tell them.”” C15  **Sample quotes relating to Table 4 in main article:**  “… trying to keep things as familiar as possible, not to change too much. And, always try one thing at a time, I always find, you know, the condition is so complex, there are so many different things going in, it’s always best just to focus on one thing at a time. Try and change too much in one go, then both the patient and the carer usually can become confused.” HCP25 (OT, Parkinson’s Service)  “Music is another one, as well, it’s fairly common, again, you have to tailor it to the person. I’ve had people with dementia where music has been very effective in terms of calming and I would often put it on at the same time as doing a consultation because it just seemed to make the communication that little bit easier.” HCP24 (Palliative Care Physician)  “…having some short conversations with them, not bombarding them with lots of information and questions but just little, maybe say, ‘We’re going to speak for 10 minutes and we’re just going to have a chat, and then I’ll ring you again tomorrow for 10 minutes.’ Just short spells, I think, sometimes are helpful to people […] medication can play a big part in their processing of information and everything, so I think the first thing you need to do is to make sure that they're at their very best when they're speaking to you.” HCP12 (PUK Adviser)  “And, it’s trying to pick out little bits in their personality is a way in, so whatever their occupation was, whatever their interests are… then you lead with the conversation that is familiar and people that are familiar, and that might give you a little bit of calm, a little bit of trust, and allow you to delve a little bit further. And, you often do get a response, even if the cognitive impairment is quite advanced and someone is not able to communicate in any normal way, you still sometimes get a bit of response if you’re leading with those techniques.” HCP24 (Palliative Care Physician)  “… let the person with Parkinson’s dictate the pace.” HCP9 (Psychologist)  “…having an understanding of what the patient’s difficulty is. So, for example, a patient with information processing difficulties may come across and say, ‘yeah, I would like that’, but actually they haven’t really processed that and are just giving you the answers that they think you want to hear.” HCP3 (Dementia Nurse Specialist)  “But I think it’s just to be open and honest about their condition, and letting them know of any aspects of it.” 15  “Whereas if you’ve got images, I think can sometimes help somebody. And keeping the information linguistically simple, short, I suppose pausing when you’re giving information, asking people to reflect on what you’ve said, giving that information back to you.” HCP6 (SLT)  “So, it’s just about taking time to explain short, concise sentences or rephrasing them or repeating them… It’s just about taking care and taking your time, not waffling on; short, concise kind of responses or conversations.” HCP7 (Nurse, Memory Service)  “But you have to give time in that instance, and you have to be incredibly patient… knowing that they might take ten, 20, 30 times as long as somebody else to respond to a question. Just knowing that rather than getting frustrated” HCP24 (Palliative Care Physician)  “I get furious, quietly furious, when they say, “Did he understand?” Now they don't know, and it may seem a fair question, it will seem a fair question to them. But when [PwP] said yes, they don’t take that as read, they then defer to me… I know you [PwP] don’t get fussed and I tend to get more fussed when they treat you [PwP] like an idiot” C11  “And because he’s trying to think of so many things at once, he gets quite agitated. But I think you've gone in on the right approach, he’s not been asked the questions, you've been explaining them and that’s quite a good thing to explain first rather than start firing straight away with questions. I think that will calm them down a bit… Maybe explaining to them that you understand that you might have difficulty communicating and just don’t worry about it. That sort of thing might put them at ease.” C6  “if someone’s having difficulty remembering something or they’re having difficulty with the word finding, can you help them, ‘OK, we’ll stop, let’s think, I’m trying to help understand what it is that you’re trying to tell me, I’m struggling at the minute. I want to understand what it is you’re trying to tell me, was it something about what we were talking about, was it something about, is there anything around the room that can help remind you’. And, if they’re really struggling and becoming quite anxious saying, ‘OK, well that might come back to you later, we’ll leave it for now’ and going onto something else, so that it doesn’t just create an anxiety cycle” HCP9 (Psychologist)  “…actually right from the start maybe separating the needs of the carer from the needs of the cared-for person” C12  “I think you’d probably have to arrange a separate time with the carer. And we do try to do that sometimes, where maybe I’ll be seeing the patient, and the OT will spend time with the carer while they’re apart. The carer can just pour out how they really feel, while I’m getting the other person to do their exercises.” HCP27 (Physiotherapist) |
| **Diagnosis of Cognitive Impairment in PD** | “No, it would usually just be things like ‘query dementia’, ‘query memory loss’, ‘query cognitive’. It’s often not been diagnosed… people interact with somebody and realise they’ve got difficulties, so it then starts to get labelled and put on medical records, and then it becomes this kind of self-perpetuating sort of story, rather than people having gone to a clinic and being given a diagnosis” HCP6 (SLT)  “because it’s [dementia] – it seems to have not been – it’s been to – have been sort of like underneath, sort of like diagnosed underneath, and nothing’s really come out of it, or anything has been said about it, or any information.” C15  “When he’s been to the Parkinson’s centre, he’s occasionally been assessed for cognitive ability. And that, he actually quite clearly showed, quite a big deficit in things like mental arithmetic and anything noticing patterns, visual patterns I think. I can’t remember everything they did […] So I think it has been established, he’s got some score on some piece of paper somewhere [laugh]. No but so I - that was a test. It wasn’t a clinic. I’d be interested in hearing about a memory clinic.” C2  “No. I turned it [cognitive assessment] down. […] I think, at the time, I was afraid I was getting worse, and I feared what was going to happen if they found – which I suspect they would find – early stages of dementia.” P13  “[we had-] a psychiatrist to come to our clinic one session a week for a year. You know, she found unmet need the clinic, she found that there was dementia we weren't explicitly diagnosing… So, she was pointing out some of these flaws, and also reminding us to, you know, refer people for living well with dementia services or courses” HCP17 (Geriatrician)  “[dementia diagnosis] can be very clinical when it’s made under a medical clinic but, actually, when it’s made under a psychiatry it’s a bit more holistic.” HCP21 (Psychiatrist)  “Whereas I think in the memory service, again without being too critical, it’s a case of you go in, you get your CT head scan, you get your bloods done and you get your ECG and you pop out with a diagnosis of dementia or mild cognitive impairment and suggestion to try Donepezil for a month and then ... So it doesn't seem as robust a service“ HCP13 (GP)  “I mean, they will be referred the question is where and how long that will take, and sometimes it’s quite long.” HCP16 (PDNS)  “So, in the community we tend to refer to the memory clinic for that diagnosis, and once they’ve got that diagnosis it tends to open up more support services for them… Day centres, if you’ve got a dementia diagnosis you will get a more appropriate day centre rather than a generic day centre, you are more likely to be signposted towards a dementia friendly day centre. It opens up a different OT, you get an OT assessment with an OT who is used to looking through things through dementia patient’s eyes rather than a generic physical disability OT” HCP22 (PDNS)  “Really integrating, probably from the very early stages, that it’s a normal part of managing your condition to talk about your emotional wellbeing and to talk about your cognitive health” HCP9 (Psychologist)  “Some people will refuse a referral to the memory clinic and most people I find accept it because they can see there is a problem or the families will support it. Often, I find I’m mentioning it and not doing anything straight away but coming back to it at a future visit.” HCP22 (PDNS) |
| **PwP & Caregivers Feeling Left in the Dark** | “…I opened it and read it, I thought I don’t really understand this.” P4  “...giving me bits of paper isn’t helpful. You get given them; you have a quick flick through them. I’ve got a folder this thick of bits of paper and information, and you’re tempted to go online and a lot of stuff’s American, which of course goes on about this drug and that drug, and of course it’s not appropriate, and their healthcare system’s different. So, there’s a lot of information out there, but it’s very scary stuff and they do always say when you’re first diagnosed with any disease, don’t look online because you’ll scare the shit out of yourself, excuse my French… You want to know, but there’s such a plethora of stuff out there that you can’t begin to sift it to be tailor-made for you. And that’s the thing with Parkinson’s, of course. Parkinson’s is an individual disease... I think you need to not just assume that you can just hand out bits of paper or information and send people away with it. It doesn’t feel comfortable. It doesn’t feel helpful.” Caregiver12  "...nobody was really very helpful in explaining the different types of dementia.” C10  “I find it hard that, if you don’t know where to go and look for information, or nobody tells you specifically what you need to do, you don’t always find it. […] I just needed somebody at the very beginning to say OK, this is what happens. Like, if you get social care help, and if you want to do the CHC, this is who you need to go to, to apply. Be it a GP or a social worker or hospital, something like that. […] It’s just, being forewarned is forearmed. And because I’m not knowing everything and I’m having to learn as I go along – so, it would’ve been helpful if somebody phoned up or come around and just said, “Right. If you need this, this is where you go, and this, and this is where you go.”” C14  [asked how she found talking about cognition with her specialist:] “I want to know more – more about it, if there was anything more I should know about it, but I didn’t get any more. Than what were saying I – what I knew before, just – I didn’t get any new ones or any different ones.” P15  “it’s like the doctor giving you pills the whole time, and just giving you more pills” P9  “nothing’s been explained, you're sent off with Parkinson’s and the tablets and then it progresses, no one knows the next day… But no, no real answers, it’s just always take you off that on, put you on that one, there's no real information on it at all really… I think it’s quite a big disease, it’s very affective on everyone in the family really. You don’t get any sort of information on it, I think you just learn it as we’re going along and it takes a big shock to start learning because something’s failed or it’s gone wrong, you know. Which is quite a sad thing.” C6  “No, I haven’t been satisfied that I had enough information. I think it’s moreso because I wanted to have a Parkinson’s nurse, because then you could ask – I could’ve spoken to her many a time when I was having a bad bout” P9  “Yes, I mean, that’s the problem, there’s a massive unmet need. Yes. I mean, I think on so many levels, on a social level there’s the carers, because it’s really tough on them, even ones that have got, sort of, wide family support struggle. And, sort of, yes, I mean, information gap, even though Parkinson’s UK and Lewy Body, they’re all trying, I think there’s still a massive gap.” HCP19 (Neurologist)  “They’re [Parkinson’s Charity] quite helpful. They tell you other things or what other people is experiencing, and all that… Yeah, and reading about it, and then you know other things that are happening around.” P15  “sometimes carers don’t really get enough time to vent their feelings” HCP12 (PUK Advisor)  Regarding peer support: “No, I think it’s important for folks to get together. Because when you do exercise, and then you have half-an-hour to have a cup of tea and a biscuit. And you say to, ‘I’ve tried this and this is working’ ... and to know what people actually feel.” P9 |
| **Falling through Gaps** | “There’s no holistic approach to anything; it’s all silos. It makes me so mad.” C12  “They [GP] don’t always appear to get the letter [from specialist], or it gets filed and I don’t think the doctors ever see it. I don’t know, because my mother’s in a group practice.” C10  “A lot of my service is geared up for people with Alzheimer’s disease, and potentially a bit of vascular.” HCP19 (OT, Memory Service)  “[Memory Services] don't have that much support to offer has been my experience. They see people once […] they’re just starting people on rivastigmine and I’m perfectly happy doing that” HCP14 (Neurologist)  “[PDNS] is really helpful, when you can get through to her, but she is so, so busy. She’s just not proactive. And she can’t be.” C10  “And I think I’m seeing the doctors, too far apart. I visit [hospital] six-monthly, and when I go there, they call me, I’m out in 10-15 minutes, so I don’t think it’s enough time for you, for a start.” P6  “The appointments, you don’t get long enough… It’s not long enough, and it’s not concentrated enough… I’m meant to have two face-to-face appointments with the consultant, each year. I don’t think I’ve had a year with two” P13  “But the practical side is that, you know, there is only one of me and I have a team of nurses and I can only see people a certain number of times a year. So, you know, as a specialist centre, how do we reach out and support people where it’s falling apart, it’s providing that support.” HCP19 (Neurologist)  “It’ll either be I ring the secretary at the GP and say, “Well, if you’re not going to do a home visit, how do I get her to this hub?” And I know it’ll be, “Well, I don’t know.” […] And if I have to phone the local hospital. But they might say, “Well, your appointment’s not with us,” you know? I don’t know where to go. And you can only Google so much, you know?” C14  “These patients need to be seen more often than we can probably see them.” HCP25 (OT, Parkinson’s Service)  “A home visit is probably the better option, because they’re in their home environment. They’re not distracted and disorientated by something strange. But that takes a whole lot more time, and you can’t get through as many patients, so.” HCP27 (Physiotherapist)  “…it really is a postcode lottery about services, and what you would expect somebody to be able to access for people with Parkinson’s it’s just not there really […] the community nurses are a far better service for people with Parkinson’s than a hospital-based nurse because a nurse can see what’s going on in their home. But of course, not every borough has a community-based nurse, so it’s quite frustrating for people when they know that other boroughs have got nurses” HCP12 (PUK Adviser)  “it is very specific to patients, but perhaps, yes, having slightly different systems where we’re perhaps more proactive” HCP8 (GP)  “But, to really manage that sort of patient I think you do need more, sort of, closer input…” HCP19 (Neurologist)  “If somebody was to say… ‘Did you know that we can offer x, y and z? And if you do, this is the number to call,’ or, ‘This is the website you can go on and book’” C14  “…when you have dementia and Parkinson’s, it’s a double challenge. So, those specific activities or groups would be really important.” HCP3 (Dementia Specialist Nurse)  “different organisations speaking to one another and accessing records” C14  “I know that colleagues in [Place] do joint clinics and they do, kind of, have multidisciplinary meetings, and that’s very much something that we don’t do locally but I think we need to start doing locally. Just having those, kind of, multidisciplinary conversations where all of the team who are involved at that particular moment have a collaborative discussion and then one person is feeding all of that back.” HCP21 (Psychiatrist) |
| **Personalising Care** | “…what one person has to deal with is not always the same for the other. That’s just my thoughts on it, that two people may not be the same.” P3  “There’s a whole range of people out there with Parkinson’s and they all need something a bit different probably, challenges. They all feel differently about it as well.” P1  “it would be nice if there was a bit of continuity, slightly more regular appointments.... If they could explain it better” C10  “So I think the other thing is you really do need to understand the condition. I think that’s the other thing. Whereas with [specialist hospital], they know the condition and they treat [PwP]… It was staggering how different it was, not with the [place] consultant, he was lovely and he is lovely, we’ve known him years. But anybody else, no idea of the condition, none, and therefore did not accommodate it. […] It’s vital that you have a relationship with them [professionals]” C11  “And carer support is very important, and specifically for Parkinson’s. Because we do have carer groups for people with dementia, but I think in terms of Parkinson’s it’s a bit different…” HCP3 (Dementia Nurse Specialist)  “So, if somebody’s getting really frustrated with their spouse because they can’t understand a word, having just a set of phrases or a small subset of phrases just enables that strain just to reduce a little bit.” HCP20 (SLT)  “So I think it’s more about empowering them [caregivers] rather than feeding them factual information, because in a way they become care managers don’t they... So it’s about that resilience and resourcefulness skilling them [caregivers] up more than necessarily a factual education I think.” HCP1 (Psychiatrist)  “I think you’ve got to always constantly remember the Parkinson’s strategies, you know, the breaking down activities, practicing strategies, fully focused attention, all those sorts of things that we do. They still need to maintain those and practice those with assistance from their carer, so it’s always remembering that. I think the strategies with the Lewy Body patients, you know, managing stress levels, trying to distract from hallucinations, those strategies are really quite good […] it goes back to the breaking things down into different stages, break everything down, the stop, think, plan, do strategy […] keeping that calm environment, getting patients to practice what they’re doing when they are able to do that so when they become more anxious or in a more pressured situation they can then have the confidence to know that they can do it […] even with the cognitive impaired those strategies still work but you may need more education for them and their carers to, kind of, assist them to do it. Again, with the carers it’s about stepping back and prompting them, rather than getting involved and doing it for them.” HCP25 (OT, Parkinson’s Service)  “I think my dad would be interested in doing anything that improved his mental acuity or memory. If there were exercises.” C2  “I have mild cognitive impairment. It would be good if there were things that I could do and should do. As I say, I do French, I do some crosswords, I try and keep myself reasonably active: thinking about various things and doing things. But if there were other things that I could or should do it would be good to hear about them. I haven’t heard much about practical things to do. […] I think it would be quite good for someone to produce something really thoughtful and well-structured about things you can do to deal with non-motor problems, in particular the memory problems. That would be really helpful.” P1  **Sample quotes relating to Table 5 in main article:**  “[tailoring] It’s not a one-off thing; it’s a process. So that then, you give what they can cope with at that moment, and then you come back even if it’s on the phone. You then contact them again at intervals. “How helpful was that? What would you still like to know?” It’s a process and that I think would be more helpful.” C12  “[Information] needs to be plain English, it needs to be available in multiple languages, and it needs to be sensitive, sort of ethnically sensitive” C12  “Because obviously one of the biggest problems that people with memory problems have is motivation, and more often than not it’s that starting an activity… if somebody can identify something they really enjoy doing, then that’s a really good resource that people should tap into.” HCP5 (OT, Memory Service)  “I think there's a lot of cultural work in terms of support that’s needed and it needs to be so sensitive, of course, to the situation, which is really hard to do… I think you need to know local population probably. I think it’s really hard, but having people employed who perhaps understand that culture well” HCP8 (GP)  “I mean, with every patient, with every family they’re coming from they will be a different age, potentially, they could be from a different culture, different educational setting or background, profession, exposure to many things. And so, you really need to address that on the individual basis, I don’t think you can have a blanket of, that’s how you do it, you really need to tailor the assessment, the care you provide, your approach to each individual depending on all of these factors that I said.” HCP16 (PDNS)  “Um, well, yeah I suppose just giving them information but then tailoring it to that person, because if it’s not relevant to you then you’re not going to really want to take that on-board but yeah. Identifying what the issues are for that person… You know just identifying what’s important to that person in all aspects of their quality of life to be- which would be different for everybody.” HCP2 (Psychiatrist)  “…our OTs will make a lot of suggestions for memory. You’ve got to find the one that’s right for that individual. So, it’s tailoring it. So, I think what is out there is useful, but I think a tailored approach, if you could produce it – that says, “These specific things would work for you because of your particular problems” – that would probably be invaluable.” HCP27 (Physiotherapist)  “I think it would be good to include obviously mental and physical well-being. Things like social well-being, different aspects of life. Because sometimes things can be neglected, but also some things can be … They can be doing very well in those aspects and they may not realise. So, they may focus on problems. And I think if you cover broadly, they can see strength there as well, which is really important. I think because giving yourself credit that, oh, actually I’m managing this pretty well or I’m managing that well. So, it is recognised not only problem focussed, but sort of is broad.” HCP3 (Dementia Nurse Specialist) |

P= Participant with Parkinson’s

C=Caregiver participant of person with Parkinson’s

HCP=healthcare professional
